# Health Survey of Numbness/Pain and Its Associated Factors in Kotohira, Japan

**DOI:** 10.1371/journal.pone.0060079

**Published:** 2013-04-01

**Authors:** Shinsuke Inoue, Masahiko Ikeuchi, Keiko Okumura, Masaya Nakamura, Chihiro Kawakami, Tatsunori Ikemoto, Motohiro Kawasaki, Toshikazu Tani, Takahiro Ushida

**Affiliations:** 1 Multidisciplinary Pain Center, Aichi Medical University, Nagakute, Aichi, Japan; 2 Department of Orthopaedic Surgery, Kochi Medical School, Nankoku, Kochi, Japan; 3 Department of Orthopaedic Surgery, Keio University, Shinjyuku, Tokyo, Japan; 4 Graduate School of Medicine, Yokohama City University, Yokohama, Kanagawa, Japan; 5 Pain Medicine & Research Information Center, Nankoku, Kochi, Japan; Hokkaido University, Japan

## Abstract

We conducted a survey of adults in Kotohira, a town of about 10,000 people located in the Nakatado District of Kagawa Prefecture, Japan. The survey was distributed to 8184 individuals, and effective responses were received from 3863 persons (response rate, 47.2%) during the survey period. Results regarding numbness and pain showed numbness alone in 7.7%, pain alone in 7.2%, both numbness and pain in 6.0%, and neither numbness nor pain in 79.6%. Spine and spinal cord damage was reported present by 5.4%, and absent by 94.6%. Analysis using the Short-Form Health Survey questionnaire, with comparison between subjects reporting both numbness and pain in the extremities and subjects with either numbness or pain alone, showed lower scores for in Short-Form Health Survey subscales (physical functioning, role [physical, emotional], bodily pain, vitality, and mental health). Subjects with numbness alone generally reported no disability in daily life. In a secondary survey, analysis of neurological findings by specialists identified 6 cases of “pain following spinal cord damage” in which spinal cord-related pain developed in the hands or feet. This represented 0.15% of the survey population starting from the primary survey.

## Introduction

Limb (arm and leg) numbness and pain can occur not only due to spine/spinal cord disorder, entrapment syndromes, diabetes, and neuropathy causing nerve dysfunction, but also due to muscle and vascular diseases. Because individuals with numbness or pain may experience great discomfort, elucidating the underlying mechanisms and developing effective treatments are very important.

Pain is an “unpleasant sensory and emotional experience associated with actual or potential tissue damage, or described in terms of such damage” [1]. However, patients with neurological dysfunction due to spine/spinal cord disorder often first complain of “shibirekan” [2], or “numbness” in English. Numbness is listed in ICD10 section R20 “disturbances of skin sensation”; and anesthesia, paresthesia, and dysesthesia (which can clearly be defined), as well as hypesthesia and some symptoms that cannot be specified, are often referred to as “numbness.” Moreover, even when pain is also present, this is sometimes expressed as “numbness.” In particular, in refractory and difficult-to-treat diseases such as cervical myelopathy, ossification of the posterior longitudinal ligament (OPLL), and syringomyelia, as well as after spinal cord injury; limb numbness and pain (allodynia or pressure sensation in the body) is severe, pain may be resistant to treatment, and quality of life (QOL) and activities of daily living (ADLs) are markedly diminished [2], [3], [4], [5]. Therefore, in pain following spinal cord damage, with symptoms of pain and numbness, elucidating the neuropathological and pharmacological mechanisms involved and developing effective treatments are of paramount importance.

According to recent nationwide surveys [6], [7], the prevalence of chronic pain with neuropathic characteristics is reported to be 7 - 8%. However, numbness information and impacts of pain and numbness on health status are largely unknown. In addition, pain directly attributable to spinal cord damage may include allodynia, in which pain is triggered by tactile stimulation that ordinarily does not cause pain, and spontaneous pressure-like pain below the level of the damaged spinal cord. Some drugs, such as anticonvulsants, are effective in some patients, but the same treatment is often ineffective in other patients with similar symptoms. Many cases are treatment-resistant, and much remains unknown about this disease population [8].

In Kotohira where this survey was conducted, a high level of cooperation exists among the council of social welfare, welfare commissioners, women’s groups, and local liaison councils; and the area is very small (8.46 km^2^) with only a small degree of population shift. This provides conditions under which the current status of town residents can very easily be ascertained (http://www.town.kotohira.kagawa.jp/english/data/index.html). The aim of this study was to clarify the prevalence of numbness and pain and their impacts on health status in a rural community in Japan, particularly spine-related symptoms were evaluated. The present study was undertaken as part of a survey on spinal-related pain (number of patients, percentage of population, symptom characteristics) (MHLW Research) in Kotohira, a town with a population of about 10,000 located in Kagawa Prefecture, Japan.

## Results

### Primary Survey Results (Fig. 1, Table 1)

Among the 119 neighborhood associations, surveys were collected from 108 neighborhood associations (2728 households, 8184 persons), and effective responses were received from 3863 persons (47.2%). This included 2141 women (55.4%) and 1722 men (44.6%). Age was <65 years in 2124 (54.5%), 65 to <75 years (young-old elderly) in 21.8%, and ≥75 years (old-old elderly) in 23.7%. Regarding limb numbness and pain, numbness alone was present in 297 (7.7%), pain alone in 280 (7.2%), both numbness and pain in 234 (6.1%), and neither numbness nor pain in 3052 (79.0%).

**Figure 1:**
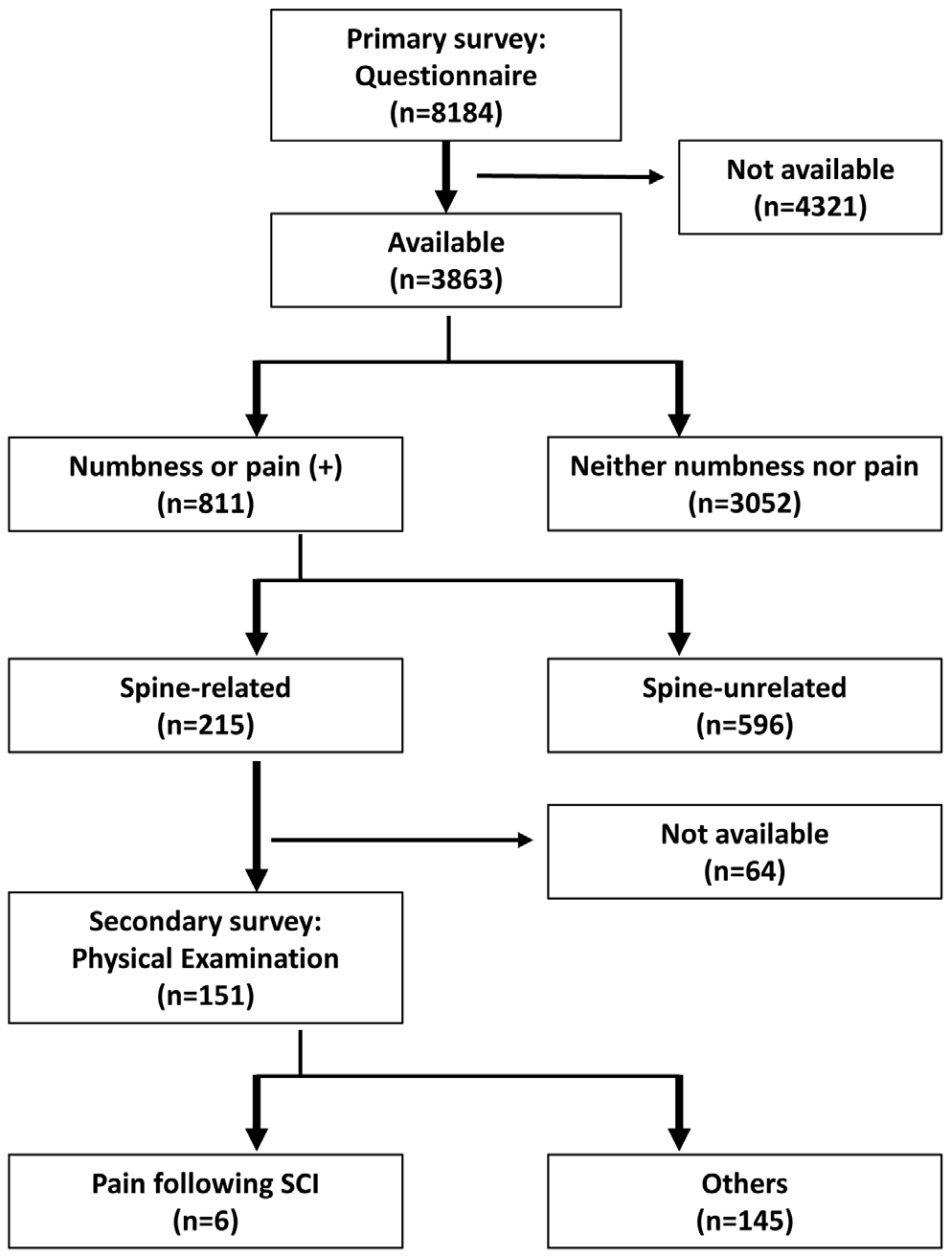
A flow diagram showing an outline of the study.

**Table 1:** Limb numbness and pain according to sex and age.

		Male	
Age	−64	65–75	75–
n	1008	358	356
Numbness+	73 (7.2)	36 (10.1)	35 (9.8)
Pain+	26 (2.6)	24 (6.7)	23 (6.5)
Both+	37 (3.7)	23 (6.4)	39 (11.0)
		**Female**	
Age	−64	65–75	75–
n	1097	484	560
Numbness+	69 (6.3)	36 (7.4)	48 (8.6)
Pain+	70 (6.4)	54 (11.2)	83 (14.8)
Both+	39 (3.6)	29 (6.0)	67 (12.0)

(Values in parentheses represent percentages).

With regard to symptoms, 215 respondents (5.6%) had been diagnosed with spine/spinal cord disorder at a hospital, while 3648 persons (94.4%) had not. In addition, 372 individuals had a history of diabetes. Taken together, the number of persons with both spinal disorder and diabetes, spinal disorder only, diabetes only, and neither spinal disorder nor diabetes was 32, 183, 346, and 3308, respectively.

**Figure 2:**
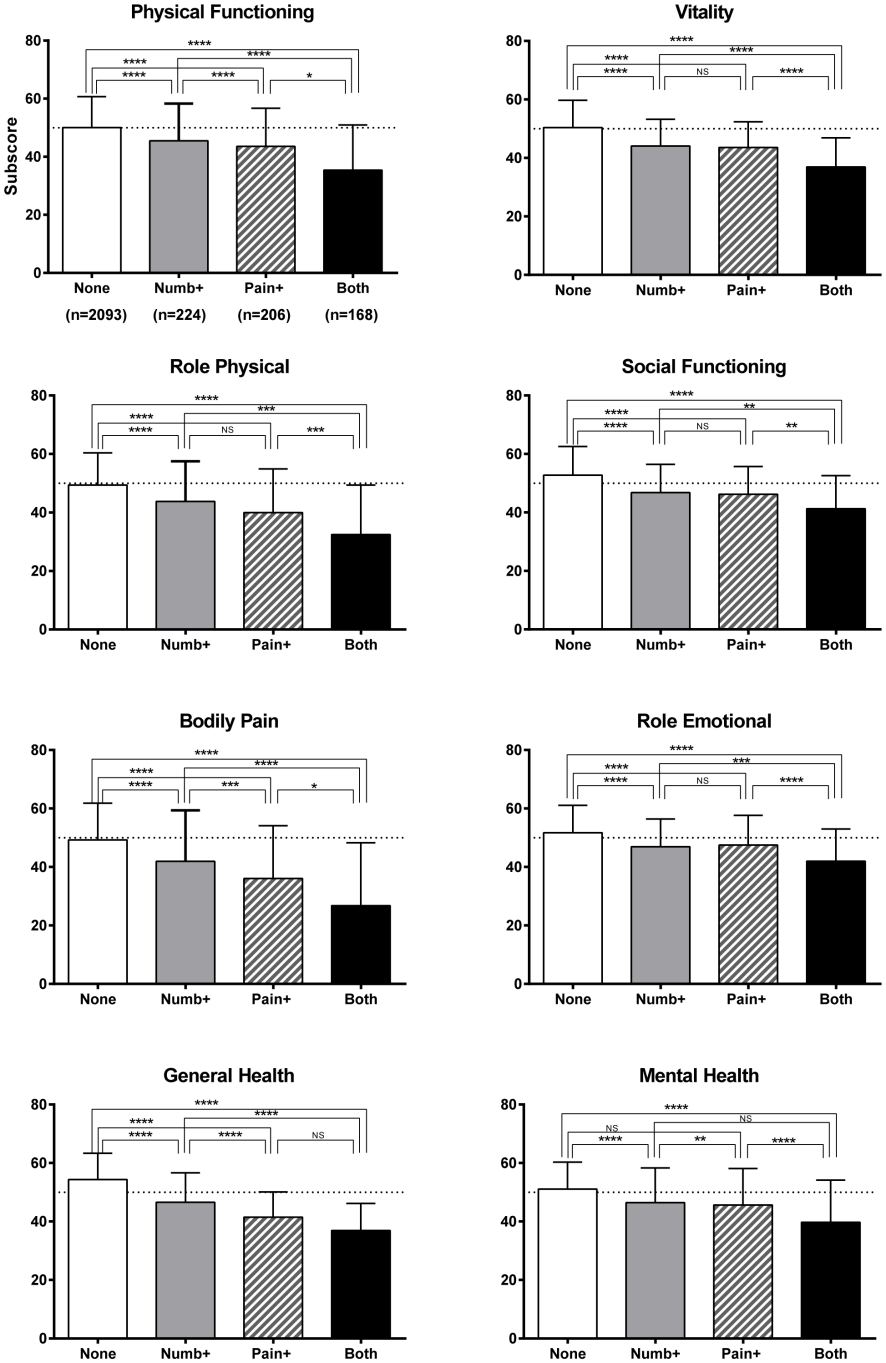
SF-36 subscale scores with presence or absence of numbness or pain. In the group with both numbness and pain, scores were significantly decreased as compared to the group with neither. *p<0.05, **p<0.01, ***p<0.001, ****p<0.0001.

**Figure 3:**
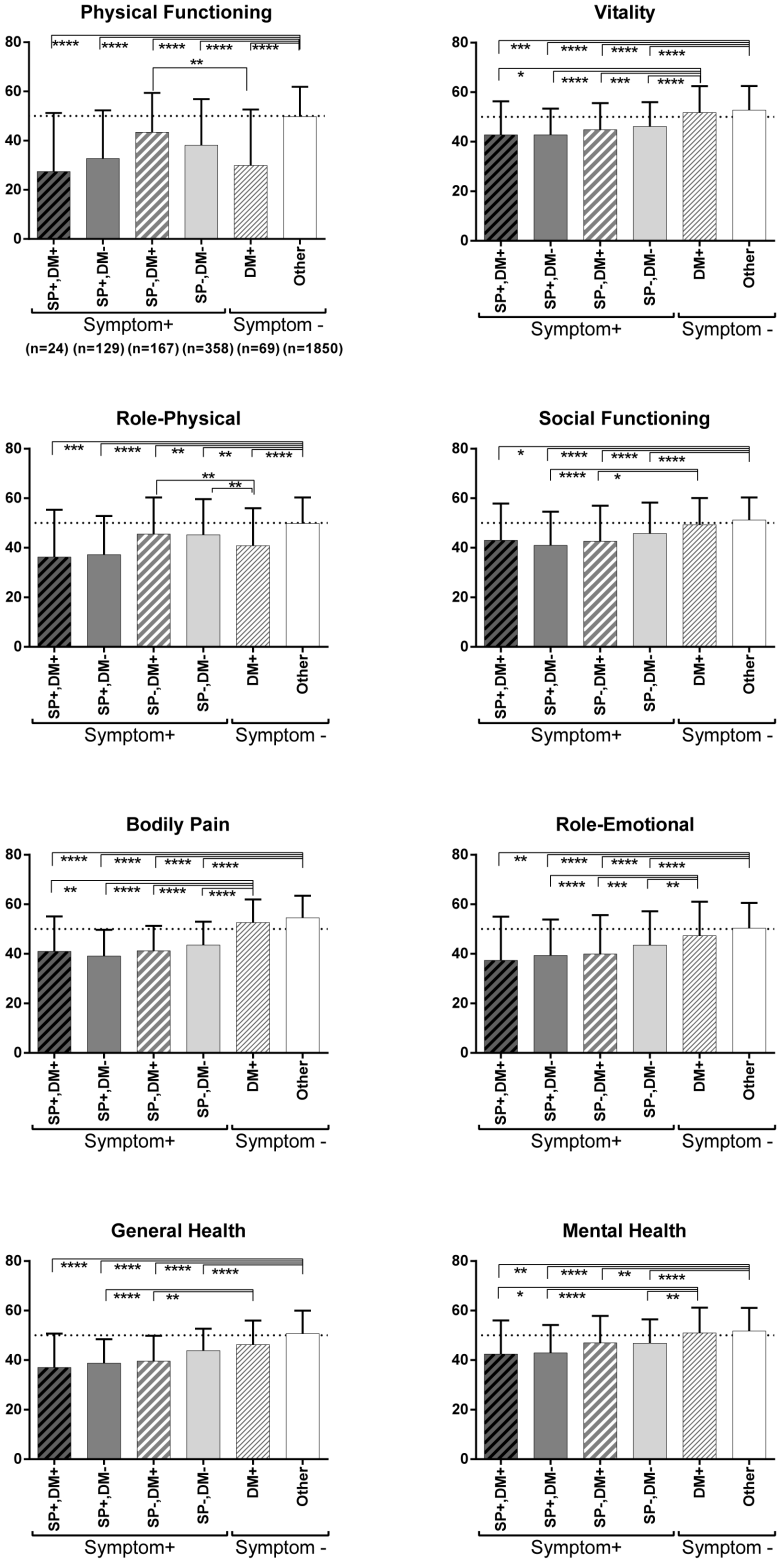
SF-36 subscale scores for presence or absence of spine/spinal cord-related disorder and diabetes. Diagnosed with spine/spinal cord-disorder (SP+), with diabetes (DM+), positive for numbness or pain (symptom +). Among individuals diagnosed with diabetes and spinal disease, health status was lower in the group with numbness or pain as compared to the group with neither. This trend was strong in those diagnosed with spine/spinal cord disorder. *p<0.05, **p<0.01, ***p<0.001, ****p<0.0001.

2691 individuals (32.8%) responded to SF-36 questionnaire. Analysis of SF-36 subscale scores showed that the group with both limb numbness and pain, as compared to the group with either pain alone or numbness alone, showed lower scores for all SF-36 subscale items except general health. Moreover, the group with either numbness or pain showed lower scores for each SF-36 item compared to the group with neither numbness nor pain. Scores for general health, physical functioning, and mental health were lower in the pain-alone group than in the numbness-alone group (Fig. 2). Among those individuals diagnosed with both diabetes and spine disease, the group with numbness or pain showed decreased health status as compared to the group without numbness or pain. This trend was stronger among individuals with a history of spine/spinal cord disorder (Fig. 3).

### Secondary Survey Results (Fig. 1)

Among those individuals with limb numbness or pain at the primary survey who had been diagnosed with spine/spinal cord disorder at a hospital, the number from whom cooperation for the secondary survey was obtained. Among the 215 residents targeted for the secondary survey was 151 persons. Based on a medical examination and a detailed interview survey in these cases, 6 individuals who have intractable spinal cord-related numbness and pain in extremities were judged to have “pain following spinal cord damage” that was resistant to ordinal treatment such as non-steroidal anti-inflammatory drugs. This represented 0.15% of the survey population starting from the primary survey. However, there were 29 persons with lumbosacral-related numbness and pain such as spinal canal stenosis or a herniated lumbar disk. In addition, cases of another cause of numbness and tingling, even though spine/spinal cord disorder had been diagnosed at a hospital, included 5 persons with limb trauma and 16 persons with arthropathy (including rheumatoid arthritis and lateral epicondylitis of the humerus).

## Discussion

Numbness is a sensory abnormality and the word is often used to describe abnormal sensations such as paresthesia, dysesthesia, and hypesthesia. Numbness is seen not only in spine/spinal cord disorder, but also often in carpal tunnel syndrome. Tay et al. reported paresthesias in 70.1% of patients diagnosed with this syndrome [9]. However, because the etiology is multifaceted with regard to the population in whom symptoms of limb numbness and pain are frequently observed, the effects of these symptoms on health status remain unclear, and thorough surveys have not been conducted to date.

In the present survey used in Kotohira, 7.7% of the population had limb numbness alone, 7.3% had pain alone, and 6.0% had both. Many individuals show symptoms, and prevalence increases with aging. One reason for an increase in numbness (a sensory abnormality caused by multiple etiologies, as described above) in older persons is that the population with spine/spinal cord disorders such as lumbar spinal canal stenosis [10], [11] and cervical spondylotic myelopathy [12], which can cause these symptoms, increases with older age. Regarding decreased sensory function, a decrease in the number of peripheral mechanoreceptors has been reported with aging, even in the absence of disease, and hypofunction [13] and myelin degeneration might be due to the involvement of such mechanisms [14]. This is thought to be linked to the mechanism by which numbness is increased in older persons. The percentage of the population of Kotohira aged 65 or older is relatively high (32%) compared with that of Japan as a whole (23%). Therefore, it is possible that the prevalence of symptoms in this study is higher than the national average.

Our survey showed that in persons with both limb numbness and pain, SF-36 subscores (physical functioning, role physical, bodily pain, vitality, social functioning, role emotional, and mental health) were lower than in participants with numbness alone or pain alone. Individuals with numbness alone generally reported no disability in daily life. However, the characteristics of numbness were not available because the questionnaire simply asked for the presence or absence of numbness in this study. It is possible that “numbness” in this study included abnormal sensation such as paresthesia and dysesthesia. A more detailed survey that can be linked to development of treatment for numbness may thus be necessary.

In a previously conducted cohort survey overseas, in all SF-36 domains except mental health, health status was impaired in the diabetes group compared to healthy persons [15]. In our survey, SF-36 subscale scores were markedly decreased in participants who had been diagnosed with spine/spinal cord disorder. In diabetes as well, when there was limb pain, each of the subscale scores tended to be decreased. This demonstrates the importance of maintaining locomotor function to control medical diseases such as diabetes and prevent chronic pain. Future development of intervention strategies to promote health status is needed [16].

Spinal cord-related pain, as pain caused by direct damage to the spinal cord, and the effects on ADL associated with this pain represent conditions caused by many diseases. Because of difficult-to-treat symptoms, treatment strategy is challenging even at facilities specializing in spine/spinal cord disorder. Causative disorders include not only spinal cord injury, but also a wide range of a smaller number of cases such as compressive myelopathy due to OPLL, syringomyelia, and spinal cord tumors. Ascertaining the whole clinical picture may thus be difficult. In our survey conducted in about half of the population of Kotohira, the data showed 6 such cases (0.15%) among about 4000 adults. The population shift in Kotohira is small, and cooperation between the town, council of social welfare and neighborhood associations is high. In this area, neighborhood associations function with support centering on the council of social welfare. Because patients with spinal cord injury usually need social support, it is unlikely that we missed a certain number of patients with severe pain related to spinal cord injury.

Mechanisms of numbness and pain in spinal cord-related pain syndromes include: 1) damage at the dorsal root level [17]; 2) damage to the dorsal horn (synapse region) (including effects of inhibition and facilitation of propagation, sprouting, and glial activation) [18]; 3) damage to spinothalamic tract [19]; 4) damage to descending inhibition pathways [20]; 5) muscle pain due to nerve damage [21]; and 6) psychosocial factors together with brain memory mechanisms [22]. To further analyze these neuropathological mechanisms and develop new treatments, a network must first be established to collect these types of patients.

## Methods

The study was conducted in cooperation among the Kotohira Council of Social Welfare, the Federation of Neighborhood Associations comprised of neighborhood association presidents, Kotohira Women’s Association, welfare commissioners, and Kotohira Town Office. All participants gave their informed written consent to the study. The requirements of data protection and medical professional secrecy were respected by all study investigators. All consent and protocols for both primary and secondary surveys had been specifically approved by the ethical committee of the Aichi Medical University.

### Primary Survey

The survey questionnaire, through the Kotohira Council of Social Welfare and Federation of Neighborhood Associations, was distributed and collected by neighborhood association presidents to 119 neighborhood associations in Kotohira Town using the placement survey method. For areas where distribution was difficult, the council of social welfare officers, welfare commissioners, and the women’s club provided assistance. Because the study would be hindered if persons in charge of distributing and collecting the surveys were unable to explain the survey, opinions of the Federation of Neighborhood Associations were sought during the stage of questionnaire creation to enable the survey to also be conducted among elderly persons. The questionnaire included items about limb numbness and pain, history of spine/spinal cord disorder, a history of diabetes, and the Short-Form Health Survey (SF-36). Because it simply asked for the presence or absence of symptoms and disease history, details of symptom and disease severity were not obtained from the primary survey. The surveys were distributed beginning on January 21, 2010 and collected by March 3, 2010.

For survey results, national standard norm-based scoring (NBS) was used for the data obtained from the SF-36. The results, including physical functioning, role physical, bodily pain, general health, vitality, social functioning, role emotional, and mental health, were analyzed using the Kruskal-Wallis test. Items with significant differences were examined with Dunn’s multiple comparison test.

### Secondary Survey

Among respondents to the primary survey with limb numbness or pain and who reported previous diagnosis of with spine/spinal cord disorder in a hospital, in those from whom cooperation was obtained, a secondary survey was conducted by specialists in spine/spinal cord disorder or neurological diagnosis. This survey was conducted as a secondary screening, or for non-participants in screening, by a telephone interview, to obtain detailed neurological findings. These patients were narrowed down to cases of refractory spinal-related pain following spinal cord damage based, when necessary, on the results of specialist examinations and imaging studies. The secondary survey was conducted from August 2010 to December 2010.
